# Dealing with RV‐oversensing; separate sensitivity settings for brady and tachy sensing

**DOI:** 10.1111/jce.14823

**Published:** 2020-12-07

**Authors:** Pranav Bhagirath, Kyle Beunder, Vokko van Halm

**Affiliations:** ^1^ Department of Cardiology Amsterdam University Medical Center Amsterdam The Netherlands

**Keywords:** CRTD, noise, oversensing, securesense, sensitivity

## Abstract

An 81‐year‐old male with a history of systolic heart failure due to an underlying ischemic cardiomyopathy with a left ventricular ejection fraction of 13% and QRS duration of 130 ms had undergone an uncomplicated cardiac resynchronization therapy defibrillator implantation (Quadra Assura MP, St. Jude Medical, LV lead (SJM Quartet 1458Q‐86), RA lead (Biotronik Safio S53) and RV shocklead (Biotronik Linox Smart S65 ProMRI) in 2015.

## 1 CASE SUMMARY

**Figure 1:**
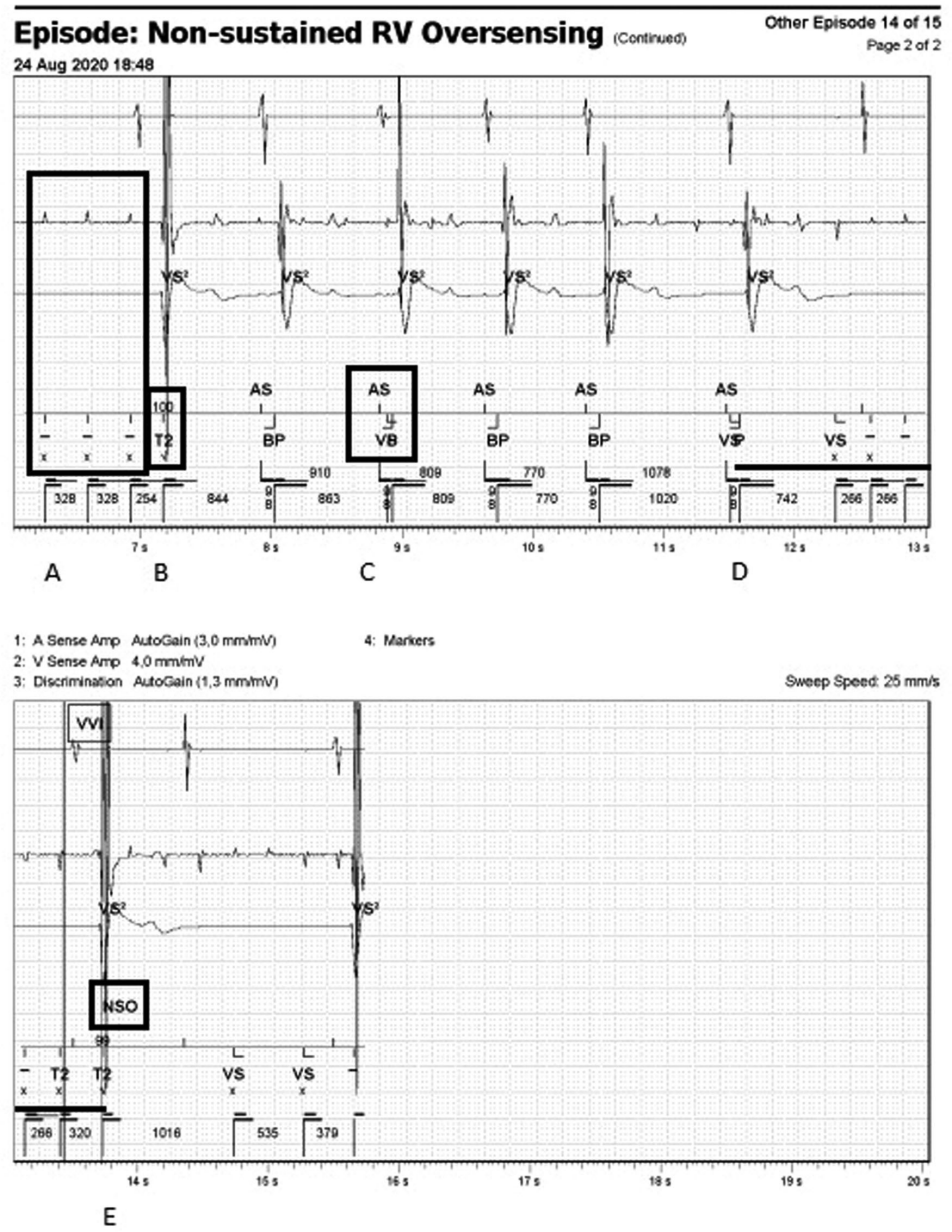
Programmer read‐out depicting repetitive noise on the ventricular lead in the VT2 zone. Device EGM with atrial sense (top panel), ventricular sense (middle panel) and discrimination channel (RV coil to can, bottom panel above the marker channel). The displayed EGM shows atrial sense followed by biventricular pacing (BP). During inhibition of RV pacing due to non‐sustained oversensing the EGM shows an underlying high degree AV block. First three RV markers (A) are unbinned non‐matching intervals indicated using “–” and X markers. The fourth marker (B) shows a 100% morphology match binned interval in the VT‐2 zone. The next markers show atrial sensing (AS) and biventricular pacing followed by a set of markers (C) showing atrial sensing (AS) followed by overlapping VS and VP marker. These markers overlap due to the different sensitivity settings programmed for bradycardia and tachycardia. Another example of overlapping VS and VP is shown after two AS–BP markers (D). This is also the start of an asystole of 2.2 s due to inhibition of ventricular pacing because of sensing the noise signal and binning it as intervals in the VT‐2 zone. (E) After a couple of seconds the SecureSense algorithm declares non‐sustained oversensing (NSO) by comparing the near field signal with the far field. EGM, electrogram; RV, right ventricular; VP, ventricular paced; VS, ventricular sensing

An 81‐year‐old male with a history of systolic heart failure due to an underlying ischemic cardiomyopathy with a left ventricular ejection fraction of 13% and QRS duration of 130 ms had undergone an uncomplicated cardiac resynchronization therapy defibrillator implantation (Quadra Assura MP, St. Jude Medical), left ventricular (LV) lead (SJM Quartet 1458Q‐86), RA lead (Biotronik Safio S53) and RV shocklead (Biotronik Linox Smart S65 ProMRI) in 2015. During follow‐up, T‐wave oversensing (TWOS) was noticed. To avoid issues related to TWOS, the bradycardia sensitivity was adjusted successfully and TWOS was not seen afterwards. In 2020 the patient was evaluated on the implantable cardioverter defibrillator (ICD)‐outpatient clinic because of (near‐)syncope. Recent home monitoring reported non‐sustained oversensing (NSRVOS) in the VT2 zone (Figure 1). At the time, the device was programmed to DDDR mode with SecureSense algorithm switched on and lower rate of 50 beats per minute (bpm) and maximum tracking rate of 130 bpm. The pacing threshold of the right ventricular (RV) lead was 0.5 V at 0.5 ms with a sense of greater than 12 mV. The pace‐sense impedance was 590 Ohm and high voltage impedance was 86 Ohm. The tachycardia therapy settings were; monitor zone 150 bpm (incount 20 intervals), VT2 zone 187 bpm (incount 30 intervals), and VF zone 240 bpm (incount 30 intervals). Triggered LV‐pacing was switched off and ventricular noise reversion mode was VOO.

The NSRVOS was caused by a stable signal with a frequency of about 3 Hz. In retrospect the same signal was detected a total of seven times over a 4‐year period. The longest episode lasted 4 min. The oversensing was detected at different moments during the day and the patient could not recall an clear explanation for the interference. After investigating the reason behind NSRVOS, it was determined that this was a non‐physiological signal with a fairly stable amplitude around 0.6 mV and with a maximum of about 0.9 mV. Previous entries of the remote monitoring revealed several episodes showing the same signal with identical frequency and amplitude. This signal was detected by the ICD resulting in the start of incount for tachy therapy and inhibition of brady pacing resulting in an asystole. So the question arose: how can we protect both brady and tachy therapy in this particular case with recurrent noise with a stable signal amplitude?

## 2 DISCUSSION

Device interrogation showed stable overall capture thresholds and impedances. Lead dislocation was excluded by means of chest X‐ray showing normal position of atrial and ventricular leads, fully similar to previous X‐rays. These findings support the hypothesis that the found interference signal was of external origin.

Upon closer examination of the ICD‐interrogation showed a few instances of different interpretations of the interference signal by the ICD (Figure 1).

The main concerns that remained were:

(1) (Near‐)Syncope caused by asystole/bradycardia due to inhibition of pacing.
(2) Oversensing induced inappropriate tachy therapy or even shocks.

The chosen solution was programming separate levels of sensitivity for the detection of bradycardia and tachycardia providing different responses to the same noise signal. The sensitivity threshold for bradycardia detection was elevated to a level above the amplitude of the recurrent noise signal (i.e., 1 mV). This significantly reduces the risk inappropriate inhibition of ventricular pacing resulting in asystole and danger of fainting. In addition, the sensitivity threshold for tachycardia detection was kept below the amplitude of the noise signal. By doing so the noise could still be monitored for change in amplitude or duration. And raising the sensitivity threshold for detecting tachycardia would also have introduced the danger of undersensing ventricular arrhythmias, especially ventricular fibrillation.

In this particular case, the SecureSense algorithm was successful in distinguishing noise from true high rate ventricular episodes and thereby provided the ability to withhold tachycardia therapy in the presence of the recurrent interference signal if it were to last longer. The SecureSense algorithm is designed reduce inappropriate therapies by distinguishing noise from true VT/VF episodes and provides the ability to automatically withhold tachycardia therapy in the presence of lead noise.^1^, ^2^ The algorithm discriminates compares the near‐field channel (V Sense Amp or RV tip to RV ring) to the far‐field (“Discrimination”) channel (RV coil to Can/RV tip to Can). If fast events are present on the near‐field channel and absent on the far‐field channel, therapy is withheld. If binned events are similar on both channels, therapy is delivered.

To further reduce the risk of inappropriate shocks the incount for ventricular tachycardia could be increased to 50 intervals. Although, increasing the incount in the VT2 zone does reduce the risk of inappropriate therapy, it could result in failure to treat life‐threatening arrhythmias due to undersensing during polymorphic VT/VF.

## 3 CONCLUSION

Recurrent noise with a certain stable signal amplitude can be dealt with by programming different levels of sensitivity for tachycardia sensing and bradycardia sensing. This could result in a remarkable device interrogation with both a ventricular sense and ventricular pace marked at the same moment. Furthermore, this separate interpretation by the ICD of the same signal can solve the interference by external noise.
